# Altered neurophysiological responses during empathy for pain in insomnia: evidence from an EEG study in non-clinical samples

**DOI:** 10.1186/s40101-023-00351-2

**Published:** 2024-01-03

**Authors:** Siyu Li, Meiheng He, Li Lin, Qingwei Chen, Taotao Ru, Guofu Zhou

**Affiliations:** 1 School of Psychology, South China Normal University, Guangzhou, 510631 China; 2 Lab of Light and Physio-Psychological Health, National Center for International Research On Green Optoelectronics, South China Normal University, Guangzhou, 510006 China; 3 Guangdong Provincial Key Laboratory of Optical Information Materials and Technology & Institute of Electronic Paper Displays, South China Academy of Advanced Optoelectronics, South China Normal University, Guangzhou, 510006 China

**Keywords:** Insomnia, Empathy for pain, Event-related potentials, Time-frequency analysis

## Abstract

**Background:**

This study aims to investigate the behavioral and neurophysiological changes accompanying the empathy for pain among individuals with insomnia in nonclinical samples, which has been scarcely explored in the existing literature despite the deleterious effects of sleep disturbance on social behavior, and interactions had been well-documented.

**Methods:**

Twenty-one individuals with insomnia in nonclinical samples and 20 healthy individuals as normal controls participated in the study. Electroencephalograph (EEG) was continuously recorded, while the participants underwent an empathy for pain task.

**Results:**

Subjective ratings of pain for painful and non-painful images revealed no statistically significant differences between the insomnia and control groups. The painful images induced a smaller P2 compared to non-painful images in the insomnia group, whereas no such difference was revealed for the controls. Moreover, a higher power density of the alpha and theta2 bands in the posterior brain regions was found in the insomnia group compared to the control group.

**Conclusion:**

These findings suggest that individuals with insomnia exhibit altered neurophysiological responses to pain stimuli and a lower capacity to share empathy for pain. These alterations may be associated with changes in attentional mechanisms.

**Supplementary Information:**

The online version contains supplementary material available at 10.1186/s40101-023-00351-2.

## Background

As one of the most common sleep disorders, insomnia is characterized by difficulty falling asleep, maintaining sleep, early awakening, poor sleep quality, and cognitive impairment during the day [1], which is identified as a risk factor for multiple undesirable mental and physical outcomes [2, 3]. With the higher academic and employment pressures, a growing number of college students experienced insomnia symptoms and subsequently impaired physiological and psychological function. Findings from a meta-analysis study reported that the prevalence of insomnia among college students in China ranged from 13.0 to 30.3% [4].

Sleep disturbance has been well established not only disrupts emotional and cognitive functioning at the individual level [5], but also affects complex socio-emotional functioning in terms of decreasing pro-social behavior [6]. Empathy is the process by which individuals understand and share the emotions and thoughts of others, and it plays an important role in human social interaction [7]. Empathy for pain, as the most typical form of empathy, refers to an individual’s perception, judgment, and emotional response to others’ pain [8, 9], which is a so-called empathic the state of empathy. It has been previously shown that insomnia-induced sleep impairment had adverse impacts on mood [10]. Meanwhile, empathy for pain itself involves both cognitive and emotional processing [11]. It thus would expect a direct effect of insomnia on empathy for pain due to the common affective nature. However, whether and the extent to which insomnia-induced chronic sleep disturbance influences the ability to share and understand others’ emotional feelings in individuals with insomnia have been scarcely investigated.

Several previous studies have investigated the relationship between sleep and empathy. For instance, a questionnaire survey conducted by Brand et al. [12] among adolescents reported that subjective perception of poor sleep correlated with distinct deficiencies in emotional competence and empathy. Rong et al. [13] found that extended nighttime sleep duration was positively associated with the capacity of cognitive empathy, whereas sleep disturbances were positively associated with emotional empathy capacity in preschoolers. Peretti et al. [14] found that sleep deprivation induced a decreased empathic response in both emotional and cognitive empathy measures. Individuals suffering from severe sleep deprivation have been shown to have difficulties with emotion regulation and empathy [15]. Nonetheless, a few studies have directly investigated the relationship between sleep and empathy for pain. One early fMRI study reported that the core neural networks of empathy (anterior insula and anterior cingulate cortex) remained unaffected with acute sleep restriction; however, the older participants perceived the painful images as more unpleasant after sleep restriction, but it was not the case for the young participants [16]. Recently, one ERP study by Duan et al. [17] revealed that acute sleep deprivation resulted in decreased amplitude of the early components (N2, N340) and reduced power density of theta2 band, suggesting the ability to empathy for pain was impaired following sleep deprivation. The authors argued that sleep deprivation induced deficits in particular components of empathic and emotional processing. However, it is still unclear whether insomnia-induced chronic sleep loss and/or impaired sleep quality would directly regulate empathy response to pain. Considering that most of the research on sleep and empathy for pain has focused on experimental depriving nocturnal sleep to cause acute sleep disturbance, there has been a notable gap in investigating the effects of insomnia-induced chronic sleep impairment on neural oscillations related to empathy for pain in individuals with insomnia. Thus, the primary purpose of the current study is to investigate the effects of insomnia on empathy for pain and further to explore the temporal dynamics of neurophysiological changes accompanying the empathy for pain among individuals with insomnia.

Referring to the existing ERP studies on empathy for pain and emotional processing, the ERP components were particularly focused with interest on P2, N2, and LPC. P2 is mainly found in the frontal, central, and parietal areas, indicating the neuronal responses to group bias within empathy [9, 18]. Research has indicated that individuals with insomnia may show alterations in P2 waves, reflecting differences in cognitive processing of stimuli and emotional regulation. For instance, Kertesz et al. [19] found that insomnia patients demonstrated significantly smaller P2 amplitudes during audio stimulation tasks compared to good sleepers. N2 is localized in the frontal, central, and temporal regions [9], and the amplitude of N2 represents the degree of emotional sharing of empathy for others’ pain [20]. Additionally, Ling et al. [21] discovered reduced N2 amplitudes in insomniacs during the Go/No-go task, suggesting potential deficits in attentional and inhibitory control. LPC, localized in the posterior parietal lobe, represents a stage of cognitive processing and evaluation of others’ pain like that of P3 [20]. These findings highlight the potential utility of ERPs in assessing and understanding the cognitive aspects of insomnia.

In addition, previous studies have reported that empathy for pain was characterized by frequency domain-specific neural activities. For instance, both the theta-band (3–8 Hz) synchronization and the alpha-band (9–14 Hz) desynchronization were associated with empathy for pain [22, 23]. Alpha oscillations underlined the sensory qualities of others’ pain and were involved in the processing of somatic aspects of empathy for pain [24], whereas emotional sharing and regulation during empathy for pain were regulated by theta oscillations [23]. To provide the persistence of dynamic neuronal oscillations in continuous signals, the current study also aims to use a TF decomposition analysis to explore the effect of insomnia on the frequency-domain measurements of neural activities associated with empathy for pain.

Hence, the current study aims to explore whether insomnia would affect empathy for pain by contrasting the response of individuals with insomnia to painful images to those who were healthy. Furthermore, the differences in the temporal domain and frequency domain-specific neural oscillations involving empathy for painful images between individuals with insomnia and the healthy are also investigated. We hypothesized that participants in the control group responded to the painful images faster than those in the insomnia group. Participants in the insomnia group would show the decreased amplitude of the early and mid-late ERP components (i.e., P2, N2, and LPC) elicited in alternative painful processing. Meanwhile, neural oscillations involving empathic responses to painful images would also be regulated by insomnia.

## Methods

### Design

This study followed a between-within subject design with the Sleep group (between-subject factor: insomnia group vs. control group) and Painful materials (within-subject factor: painful, non-painful) as independent factors. Each participant visited the lab once, and both painful and non-painful images were administered alternately.

### Participants and screening

The expected sample size was calculated using the G*Power 3.1.6 software [25], indicating that a sample size of 17 for each group yields 80% power to detect a moderate effect size (0.25) with an error probability of 0.05. Therefore, 21 participants were initially recruited for each group, leading to a total of 42 participants. However, one participant withdrew from the experiment for personal reasons. In the end, a total of 21 participants with insomnia (7 males) and 20 control healthy participants (8 males) participated in the laboratory study (see Table 1).

**Table 1:** Demography of participants in the control and insomnia groups

	Controls (*N* = 20)		Insomnia (*N* = 21)		*t*	*p*
	*M*	*SD*	*M*	*SD*		
Male	8		7			
Female	12		14			
Age	20.7	2.01	21.7	2.82	− 0.86	0.4
Years of education	14.8	2.41	16.2	2.32	− 0.98	0.52
PSQI	3.3	1.63	11.71	2.8	− 11.7	< 0.001
ISI	2.7	2.01	12.71	2.76	− 13.13	< 0.001
BDI-II	6.89	3.13	8.23	3.57	− 2.67	< 0.05

*M* mean, *SD* standard deviation, *PSQI* Pittsburgh Sleep Quality Index, *ISI* Insomnia Severity Index, *BDI-II* Beck Depression Inventory-II

Participants were recruited for the study through online advertisements at a local university. The participants included in the control group and insomnia group were carefully matched in their age, gender, and education level. Table 1 presents the demographic characteristics of participants in both the insomnia and control groups. It is important to note that all employed participants had normal vision and had not taken any sleep-affecting medications in the month leading up to the study, ensuring a consistent baseline for analysis.

Participants in the insomnia group were screened in non-clinical samples according to the criteria of the International Classification of Sleep Disorders (ICSD) [1], which may not necessarily align with those individuals clinically diagnosed with insomnia by healthcare professionals or clinicians, including the following:

1. The Pittsburgh Sleep Quality Index (PSQI, [26]) > 7
2. Insomnia Severity Index (ISI, [27]) > 7
3. Sleep problems last longer than 1 month and occur at least three times per week.
4. The Beck Depression Inventory-II (BDI-II, [28]) < 12

The inclusion criteria for participants in the control group were as follows: (1) PSQI < 7 and ISI < 7, (2) content with their regular sleep patterns and have not experienced any insomnia symptoms in the past 3 months, (3) sleep latency < 30 min and total nighttime sleep time ≥ 7 h, and (4) BDI-II < 12. The participants employed in the control group were matched with those in the insomnia group in terms of age, gender, and education level and had none of the diagnostic criteria of insomnia met. All participants provided written informed consent, and the study was approved by the ethical committee at a local university.

### Procedure

An online screening questionnaire was sent to all potential participants 1 week before the laboratory study. Participants who met all the screening criteria were required to complete sleep diaries [29] to monitor their regular sleep quality and sleep–wake schedule. After arriving in the lab, participants were briefly interviewed and filled out questionnaires regarding their demographics, baseline mood, and sleep quality of the preceding night. Afterward, electrode caps were fitted to participants. To maintain fixation at the fixation crosses and limit head movements during EEG recording, participants were instructed to blink as little and in the absence of a stimulus as possible. The formal experiment started with a few practice trials. Then, two blocks of images were administered, with one block containing painful images and the other one containing non-painful images.

### Stimuli and empathy for pain task

**Fig. 1:**
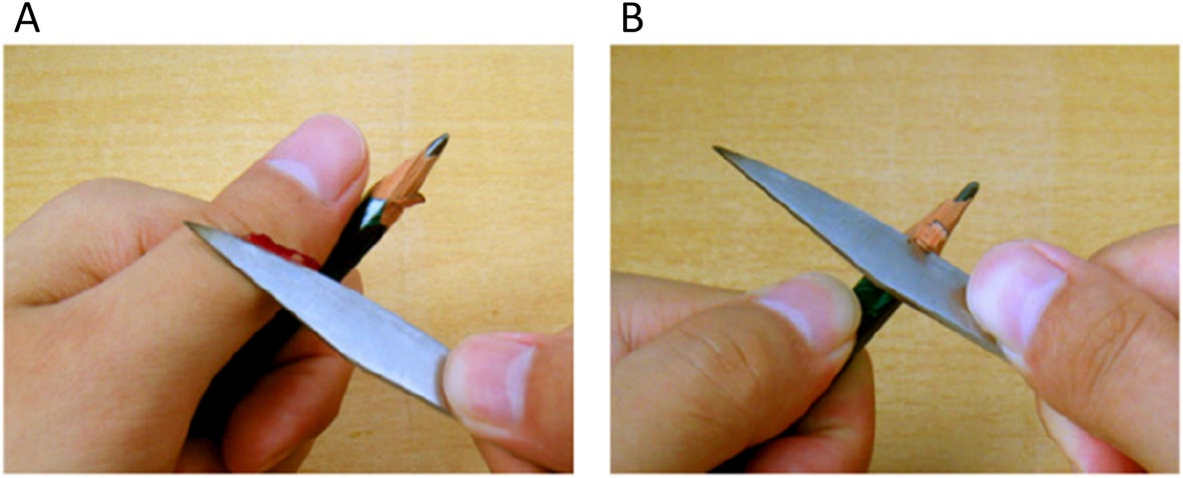
Examples of image materials used in the experiment. **A** Painful stimulus. **B** Non-painful stimulus

One hundred images (50 painful and 50 non-painful images) were selected from a previous study by Meng et al. [9]. This set of images was also widely used in subsequent pain-related studies [30–35]. The painful intensity, valance, arousal, and motion of all painful and non-painful images were subjectively assessed in a study by Meng et al. [9], suggesting the manipulations of all images were valid. The descriptive statistics of employed painful and non-painful images in the current study are shown in Table 2. It should be noted that all the images are presented in pairs, with one representing a painful scenario and the other a non-painful scenario (see Fig. 1). Each image was manipulated with the same dimensions of 9 cm × 6.76 cm (width × height) and a precision of l00 pixels per inch. The brightness, contrast, and color of the images were matched between the painful and non-painful images.

**Table 2:** The descriptive statistics of painful and non-painful images employed in the current study

	Painful (*N* = 50)		Non-painful (*N* = 50)		*t*	*p*
	*M*	*SD*	*M*	*SD*		
Painful intensity	5.71	0.59	1.76	0.46	− 39.98	< 0.001
Valance	3.41	0.35	4.50	0.49	13.86	< 0.001
Arousal	5.47	0.48	2.39	0.49	− 34.11	< 0.001
Motion	4.92	0.52	4.75	0.40	0.92	0.576

*M* mean, *SD* standard deviation

**Fig. 2:**
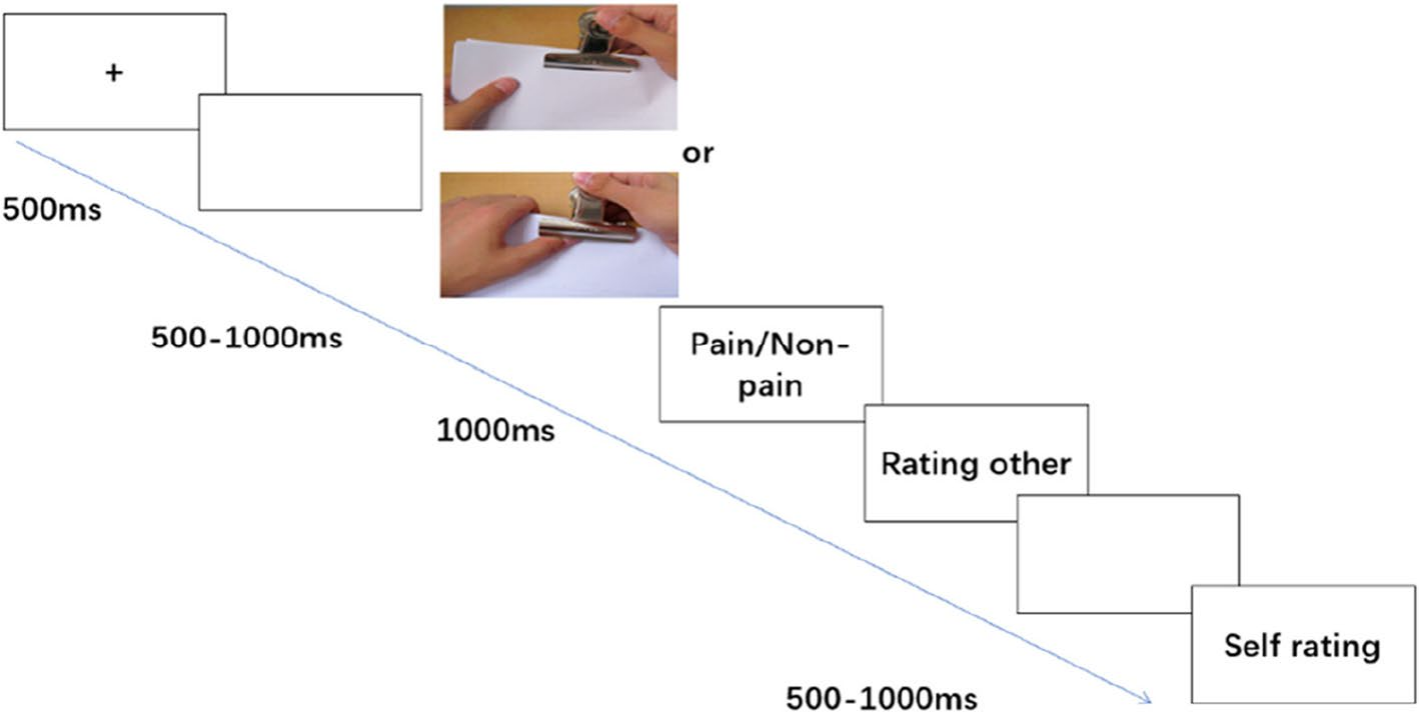
Flow chart of the experiment

Each trial started with a 500-ms fixation “ + ” on the screen center followed by a blank screen for 500–1000 ms, and then one of the paired images was randomly displayed for 1000 ms (see Fig. 2). The participants were asked to press the “F” key if they thought the person in the image was painful; otherwise, press the “J” key. Afterward, the participants were asked to subjectively rate the pain level of the person in the image on a 9-point Likert scale ranging from 1 “no painful” to 9 “very painful” and the degree of self-rating on a 9-point Likert scale ranging from 1 “no unpleasantness” to 9 “very unpleasantness”; the two slices for subjective ratings were run with a random interval of 500–1000 ms and were terminated after participants’ responses. Random intervals aim to ensure task independence and minimize biases by making the start of each task unpredictable. This helps prevent participants from forming temporal patterns or expectations. A total of 100 trials were presented in two blocks, each including half of the trials.

### Baseline sleep and mood

PSQI was used to assess participants’ baseline sleep quality. The Self-rating Anxiety Scale (SAS) [36] and the Positive Affective and Negative Affective Schedule (PANAS) [37] were employed to assess the baseline mood.

### Electroencephalogram (EEG) recording and preprocessing

EEG was collected with an 88-channel EEG amplifier (ANT EEGO; eemagine Medical Imaging Solutions GmbH, Berlin, Germany) with 64 electrodes placed on the scalp in accord with the requirements of the international 10–20 system. All channels were referenced during recording to a reference electrode positioned at CPz, and an electrode positioned at the FPz and Fz served as ground. The electrooculogram (EOG) channels for recording vertical eye electricity were located 1 cm below the left orbit. All electrode impedances were kept below 5 kΩ, and the bandpass filter was set at 0.01–100 Hz with a sampling rate of 1 kHz.

EEG data were processed offline using MATLAB (v. R2014a; the MathWorks Inc.) and with the EEGLAB toolbox (version 13.5.4b). To exclude low-frequency drift and high-frequency noise signal interference, the data is filtered using a digital zero-phase bandpass filter with a slope of 24 dB/octave from 0.01 to 30 Hz. Subsequently, the reference was converted to the average of the bilateral mastoid signals, and continuous EEG data were reference converted. Eye artifact correction was accomplished separately for each participant by subjecting the EEG data recorded to independent components analysis (ICA; [38]). Eye artifacts were removed by identifying their topography. Subsequently, continuous EEG data were divided into segments (− 200 ms before stimulus initiation to 800 ms after stimulus presentation), and baseline correction was performed using 200 ms (− 200 to 0 ms) before stimulus presentation as a baseline.

The current study is particularly interested in the neural activities involved in the ERP components, including painful stimulus-elicited early components (P2, N2) and late components (LPC). Based on visual inspection of the grand averages, scalp topographies, and previous research, electrode grand averages were calculated separately for P2 waveform (F3, F4, FC1, and FC2 electrodes), N2 (FC5, FC6, and Cz electrodes), and LPC (FC1, FC2, Cz, and FCz electrodes; 300 − 600 ms). Peak amplitude and latency were detected as local maxima and minima in certain time windows (P2: 120 − 180 ms; N2: 230 − 330 ms). Regarding time–frequency-domain indicators, the total EEG power was analyzed by following these steps, and the specific electrodes were determined. First, single-trial data were used to estimate the oscillatory power via the Morlet continuous wavelet transform (MCWT, [39]. The parameters of central frequency (ω) and restriction(σ) in MCWT were 5 and 0.15, respectively [40]. Time–frequency representations (TFRs were explored in the range of 1–40 Hz in steps of 0.5 Hz. Second, single-trial TFRs were averaged to obtain the averaged TFRs of every participant under each condition. Third, the averaged TFRs were subsequently cut in length (− 500 to 1500 ms to reduce the edge effects. Fourth, the power was normalized by conversion to a decibel (dB scale [10_ log10 (power/baseline]. The baseline power was computed as the average power across all experiment conditions, from − 500 to − 200 ms.

Based on previous studies [17, 22, 23], the current study identified three time–frequency regions of interest (TF-ROIs): the alpha-band (8–13 Hz, 100–650 ms) theta1 (2–4 Hz, 200–500 ms) and theta2 (5–7 Hz, 200–500 ms), and the density power was averaged to obtain non-phase-locked components [41]. Meanwhile, the spatial regions of interest (S-ROIs): the FC1, FCz, and FC2 electrodes, were selected and collapsed by averaging their values to obtain an indicator of anterior activity; the P1, Pz, and P2 electrodes were selected and collapsed by averaging their values as an indicator of posterior activity.

### Statistical analysis

Linear mixed model (LMM) analyses were performed to separate the effects between individuals and within individuals with R Studio (version 4.0.3) (“psych” and “plyr” packages were used for the preparatory analyses, “emmeans,” “Sjstats,” “lme4,” and “lmerTest” packages were used for the statistical analysis). In addition, the “sjstats” toolkit was used to calculate the effect sizes of the LMM models, and this study used the effect size indicator *η*_*P*_^2^ to estimate and describe the effect sizes. To account for the two levels (individual level and trial level) in the data, we used a random intercept model, that is, in addition to the fixed effects, we added a random effect with “participants” as a categorical variable. This allowed each participant to have their own unique intercept, reflecting the differences between individuals, and improved the fit and accuracy of the model parameters. The data from two participants in the insomnia group were excluded due to their excessive EEG artifacts, and the data from 39 participants were included in the formal data analysis.

First, independent-sample *t*-tests were used to verify whether there were any potential differences in baseline measures of sleep variables and mood because the baseline sleep quality and mood (anxiety and positive and negative affects) were measured once for each group. These baseline measures were considered as covariates in subsequent analyses to control for their potential effects.

An LMM analysis for accuracy rate (ACC) and response time (RT) was conducted to explore behavioral differences in empathy for pain, with “Participants” as a random intercept to cluster the data per participant, with Sleep group (insomnia vs. control), and Painful materials (painful vs. non-painful) as a fixed factor, and the interaction between Sleep group and Material was added. In the LMM analyses, to explore the group differences in ERP components, the Sleep group and Painful materials were added as fixed factors, and the P2, N2, and LPC were added as dependent variables, respectively. With respect to the time–frequency domain indicators, Brain region (anterior vs. posterior) was added as an additional fixed factor and the alpha, theta1, and theta2 band power as dependent variables. The nature of all interactions and comparisons between different pain materials was explored using Bonferroni correction.

To explore the link between neural and behavioral response to empathy for pain, the Pearson correlational analyses between time-domain indicators (mean amplitude of N2 and LPC, peak amplitude of P2) and RT and between frequency-domain indicators (alpha, theta2) and RT were performed for the two groups (control and insomnia group) across all Painful materials conditions, respectively.

## Results

### Baseline sleep and mood

The independent-sample *t*-tests revealed significant differences in the anxiety and negative mood between the insomnia and control groups (*p*s < 0.05); participants in the insomnia group showed average worse sleep quality, more anxiety, and negative mood than those in the control group (see Table 3).

**Table 3:** Baseline comparison of sleep quality and mood between the two groups

	Controls (*N* = 20)		Insomnia (*N* = 21)		*t*	*p*
	*M*	*SD*	*M*	*SD*		
Positive mood	31.1	6.42	29.81	6.36	0.65	0.52
Negative mood	17.25	6.12	21.71	7.85	− 2.02	0.04
SAS	36	5.76	47.32	9.2	− 4.7	< 0.001

*M* mean, *SD* standard deviation, *SAS* Self-rating Anxiety Scale

### Subjective ratings and behavioral performance of empathy for pain

The descriptive results of behavioral indicators are shown in Table 4. LMM analyses for RT and ACC revealed that the main effects and interaction effects did not reach significance [ACC: *F*_Group_ (1, 39) = 0.61, *η*_*P*_^2^ = 0.012, *p* = 0.44; *F*_Materials_ (1, 39) = 0.46, *η*_*P*_^2^ = 0.009, *p* = 0.50; *F*_Interaction_ (1, 39) = 2.25, *η*_*P*_^2^ = 0.04, *p* = 0.14; RT: *F*_Group_ (1, 39) = 2.59, *η*_*P*_^2^ = 0.052, *p* = 0.12; *F*_Materials_ (1, 39) = 2.29, *η*_*P*_^2^ = 0.046, *p* = 0.14; *F*_Interaction_ (1, 39) = 1.27, *η*_*P*_^2^ = 0.026, *p* = 0.27]. Painful materials revealed a significant main effect [*F* (1, 19) = 273.83, *η*_*P*_^2^ = 0.891, *p* < 0.001], with the subjective ratings of emotional pain for the painful images being higher than the ratings for the non-painful images. Meanwhile, LMM analyses for cognitive empathy revealed a significant main effect of Painful materials [*F* (1.19) = 186.11, *p* < 0.001, *η*_*P*_^2^ = 0.835], with the participants’ cognitive empathy scores for the painful images being higher than those for the non-painful images. LMM analyses for emotional empathy revealed a similar case with cognitive empathy. Painful materials also yielded a significant main effect [*F* (1, 19) = 273.83, *p* < 0.001, *η*_*P*_^2^ = 0.872], with the participants’ emotional empathy scores for the painful images being higher than those for the non-painful images. These findings suggested that participants showed more empathy for the painful images regardless of whether they were insomnia or not.

**Table 4:** Descriptive results of behavioral indicators

	Controls (*N* = 20)		Insomnia (*N* = 21)	
	Non-painful	Painful	Non-painful	Painful
	*M* ± *SE*	*M* ± *SE*	*M* ± *SE*	*M* ± *SE*
ACC	0.910 ± 0.02	0.919 ± 0.02	0.949 ± 0.02	0.925 ± 0.02
RT	820 ± 100.7	834 ± 100.7	1102 ± 97.6	1104 ± 97.6
Cognitive empathy	1.10 ± 0.34	5.07 ± 0.34	1.39 ± 0.34	5.41 ± 0.34
Emotional empathy	1.14 ± 0.34	5.43 ± 0.34	1.46 ± 0.34	5.81 ± 0.34

*M* mean, *SE* standard error, *ACC* accuracy rate, *RT* response time

### ERP results

**Fig. 3:**
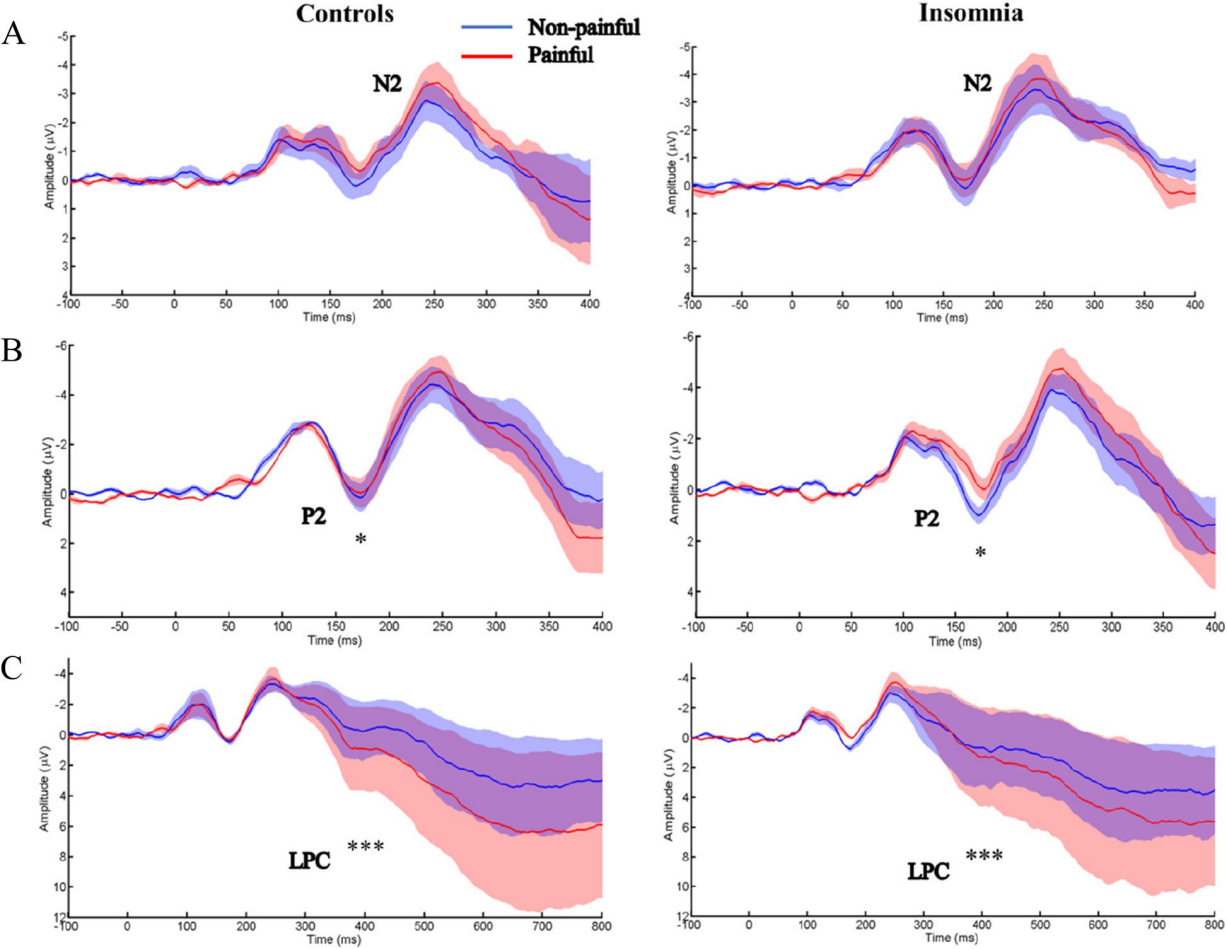
ERP waveforms for the empathy for pain task. The red- and blue-shaded areas represent the standard error of the mean (SEM); *p < 0.05. ***p < 0.001. **A** N2 component, time windows 230–330 ms. **B** P2 component, time windows 120–180 ms. **C** LPC component, time windows 300–600 ms

**Fig. 4:**
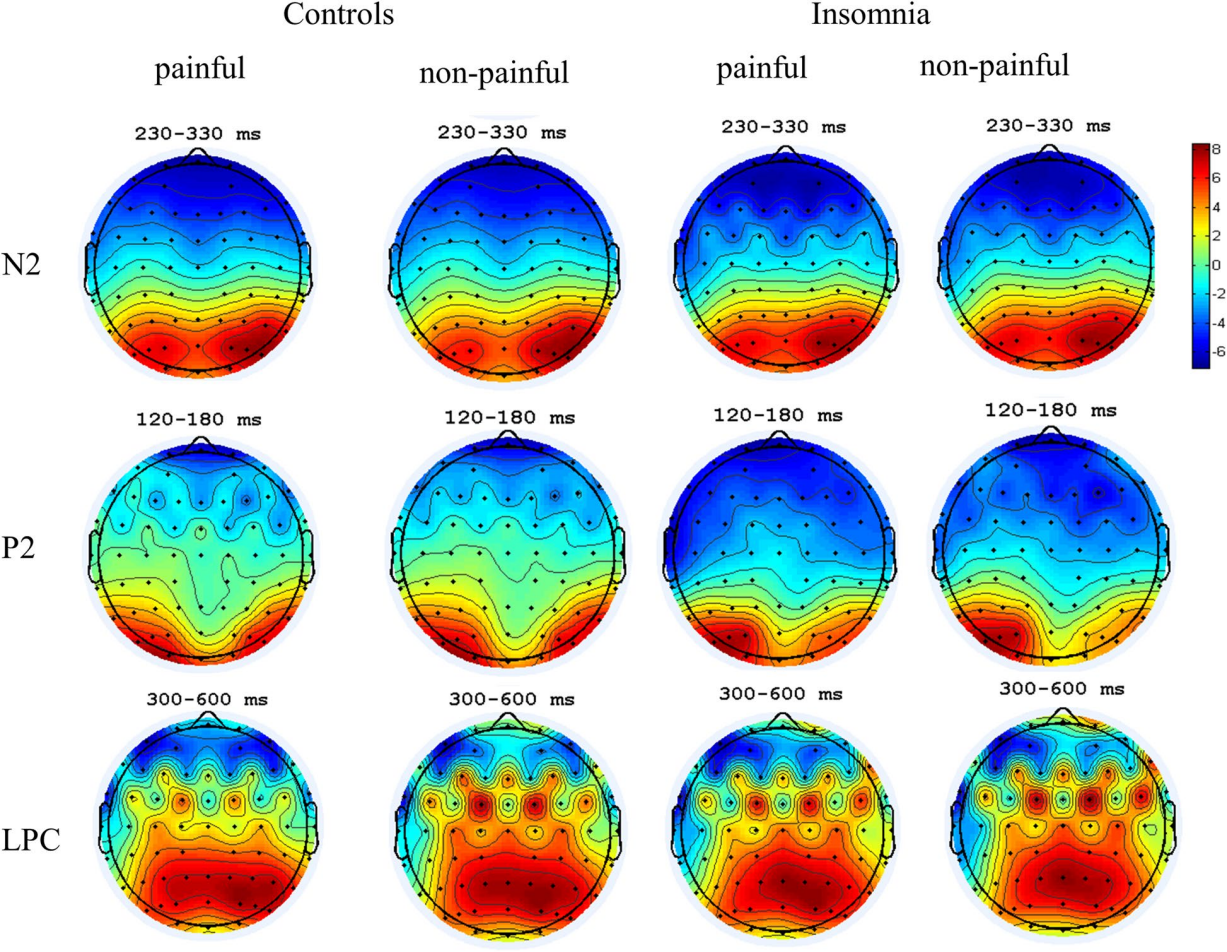
The topographies under different conditions. The control group on the left, the insomnia group on the right

The ERP waveforms and the topographies for the empathy for pain task can be seen in Figs. 3 and 4, respectively, and the descriptive statistical data is included in the supplementary materials. As for N2 peak amplitude, the Sleep group and Painful materials yielded no significant main effects (*F*_Group_ (1, 43) = 2.13, *η*_*P*_^2^ = 0.044, *p* = 0.15; *F*_Materials_ (1, 39) = 0.08, *η*_*P*_^2^ = 0.008, *p* = 0.59), and the interaction effect between Sleep group and Painful materials was not significant (*F* (1, 39) = 0.30, *η*_*P*_^2^ = 0.006, *p* = 0.59). The mean amplitude of N2 also yielded no significant main effects of Sleep group and Painful materials (*F*_Group_ (1, 43) = 2.26, *η*_*P*_^2^ = 0.048, *p* = 0.14; *F*_Materials_ (1, 39) = 0.03, *η*_*P*_^2^ = 0.001, *p* = 0.86), and the interaction effect was also not significant (*F* (1, 39) = 0.69, *η*_*P*_^2^ = 0.015, *p* = 0.41). Similarly, there were no significant main effects of Sleep group, Painful materials, nor their interaction effect on the peak latency (*F*_Group_ (1, 56) = 0.41, *η*_*P*_^2^ = 0.007, *p* = 0.53; *F*_Materials_ (1, 39) = 2.99, *η*_*P*_^2^ = 0.051, *p* = 0.09; *F*_Interaction_ (1, 39) = 1.39, *η*_*P*_^2^ = 0.023, *p* = 0.25).

The peak amplitude of P2 revealed a significant interaction effect between Sleep group and Painful materials [*F* (1, 39) = 5.51, *p* = 0.04, *η*_*P*_^2^ = 0.09]. The post hoc analyses indicated that participants in the insomnia group showed significantly smaller P2 for the painful images than the non-painful images (*EMM*_painful_ = 0.39, *SE* = 0.92; *EMM*_non-painful_ = 1.71, *SE* = 0.92, *p* = 0.02), while the amplitude of P2 elicited by painful images did not differ with that elicited by non-painful images in the control group (*p* = 0.68). No significant main effects were revealed (*F*_Group_ (1, 45) = 0.38, *η*_*P*_^2^ = 0.007, *p* = 0.54; *F*_Materials_ (1, 39) = 2.40, *η*_*P*_^2^ = 0.048, *p* = 0.13). For both the mean amplitude and peak latency of P2, no significant main effects nor interaction effects were found (mean amplitude: *F*_Group_ (1, 43) = 0.37, *η*_*P*_^2^ = 0.008, *p* = 0.55; *F*_Materials_ (1, 39) = 0.94, *η*_*P*_^2^ = 0.021, *p* = 0.34; *F*_Interaction_ (1, 39) = 2.06, *η*_*P*_^2^ = 0.046, *p* = 0.16; peak latency: *F*_Group_ (1, 56) = 3.12, *η*_*P*_^2^ = 0.054, *p* = 0.08; *F*_Materials_ (1, 39) = 0.91, *η*_*P*_^2^ = 0.016, *p* = 0.35; *F*_Interaction_ (1, 39) = 2.35, *η*_*P*_^2^ = 0.041, *p* = 0.13).

The mean amplitude of LPC revealed a significant main effect of Painful materials [*F* (1, 39) = 15.53, *p* < 0.001, *η*_*P*_^2^ = 0.25], with the painful images eliciting larger LPC than the non-painful images (*EMM*_painful_ = 3.23, *SE* = 0.70; *EMM*_non-painful_ = 1.46, *SE* = 0.70,* p* < 0.001). The main effect of Sleep group and interaction effect between Sleep group and Painful materials did not reach significance (*F*_Group_ (1, 41) = 0.20, *η*_*P*_^2^ = 0.003, *p* = 0.66; *F*_Interaction_ (1, 39) = 3.43, *η*_P_^2^ = 0.06, *p* = 0.07).

### Correlation between task performance and ERP indicators

**Fig. 5:**
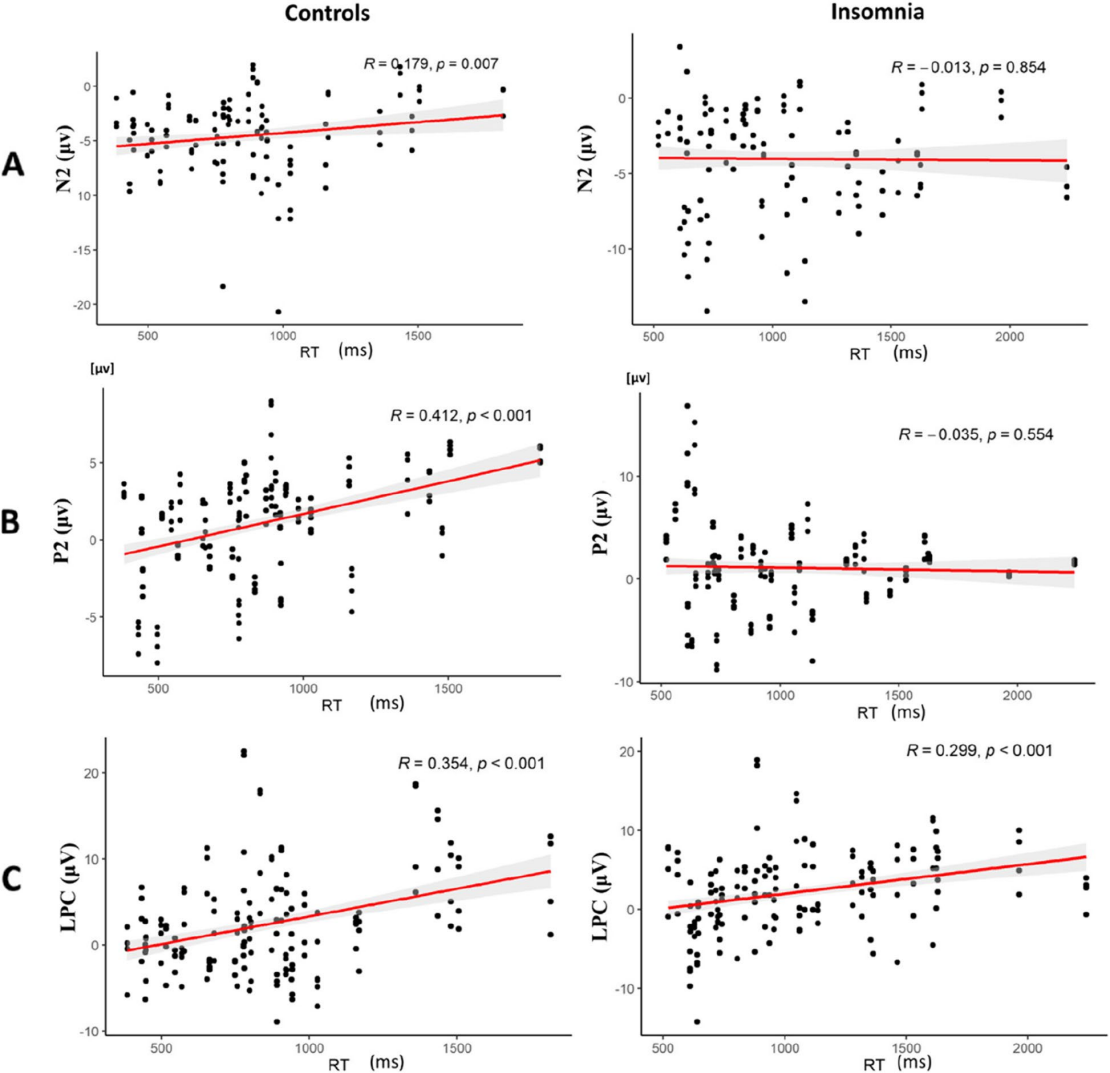
Scatter plot of the correlation coefficient between task performance and ERP amplitude. **A** Scatter plot of the peak amplitude of N2 and response time for the control and insomnia groups. **B** Scatter plot of the peak amplitude of P2 and response time for the control and insomnia groups. **C** Scatter plot of the mean amplitude of LPC and response time for the control and insomnia groups

The correlational analyses revealed that the mean amplitude of N2 was significantly positively correlated with RT in the control group across all Painful material conditions (*r* = 0.18, *p* = 0.007), while it was not the case for the insomnia group (*r* = −0.01, *p* = 0.85). The peak amplitude of P2 was significantly correlated with RT in the control group (*r* = 0.41, *p* < 0.001), while no such correlation was revealed in the insomnia group (*r* = −0.04, *p* = 0.55). Meanwhile, the mean amplitude of LPC was positively correlated with RT in both groups (*r*_control_ = 0.35, *r*_insomnia_ = 0.30, *ps* < 0.001) (see Fig. 5).

### Time–frequency-domain results

**Fig. 6:**
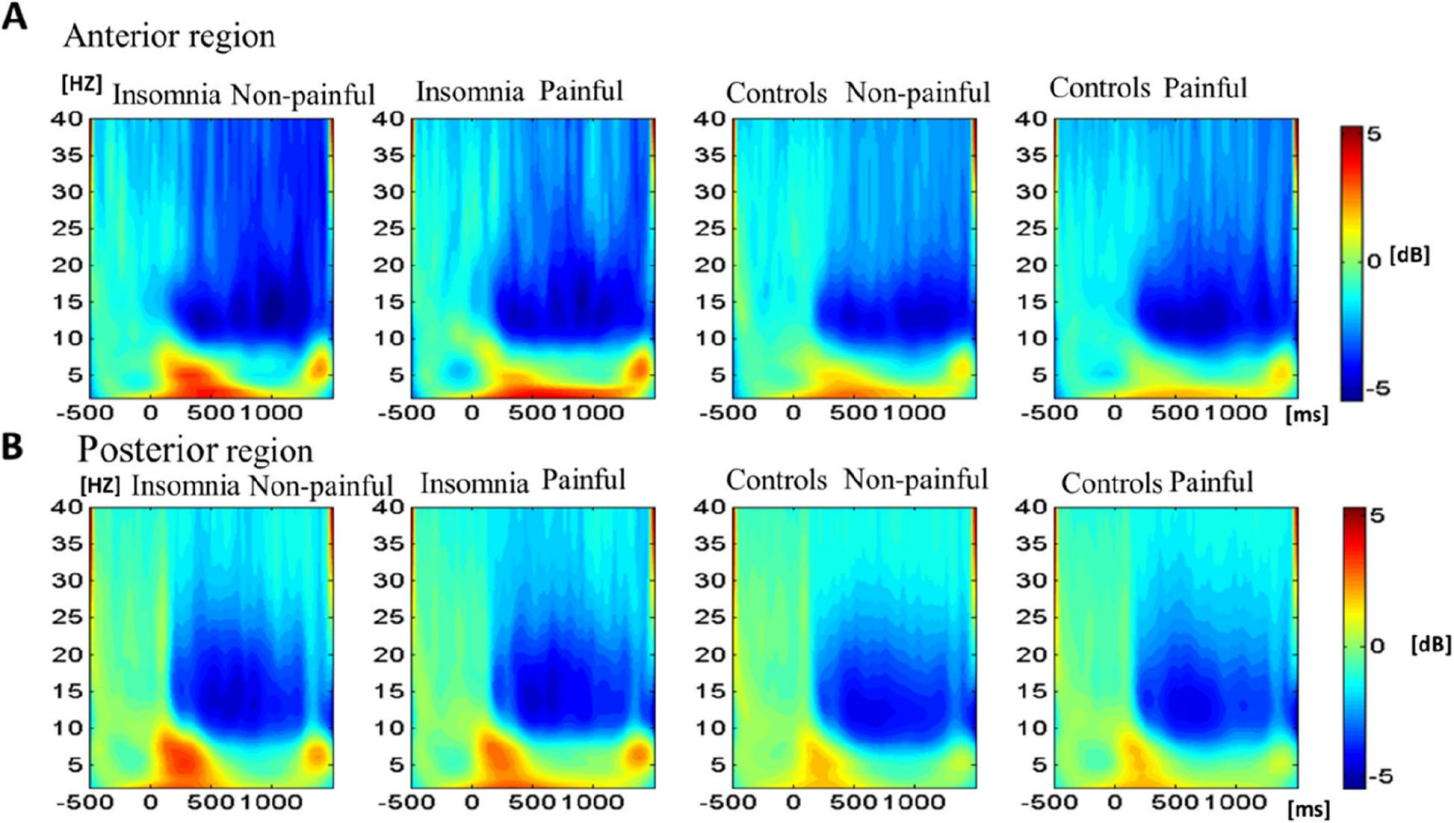
EEG power responses. **A** Grand-averaged power is produced by painful and non-painful arguments in the anterior regions (the anterior region of interest is the superimposed average of FC1, FCz, and FC2 electrode points). **B** Grand-averaged power is produced by painful and non-painful arguments in the posterior regions (the posterior region of interest is the superimposed average of P1, Pz, and P2 electrode points)

The frequency-domain analyses in four conditions (insomnia-painful; insomnia-non-painful, control-painful, control-non-painful) consistently revealed that the lower frequency band (< 8 Hz) showed the maximum event-related synchronization (ERS) at 0–600 ms, while the higher frequency band showed the maximum event-related desynchronization (ERD) at 500–1000 ms (see Fig. 6). Thus, the relative power of four spectrum evoked in these time-windows was further investigated.

The relative power of the alpha band revealed a significant main effect of Brain region [*F* (1, 117) = 46.159, *p* < 0.001, *η*_*P*_^2^ = 0.26], with the larger alpha power being observed in the anterior regions than the posterior regions (*EMM*_anterior_ =  − 0.98, *SE* = 0.30; *EMM*_posterior_ =  − 1.83, *SE* = 0.30,* p* < 0.001). The interaction effect between Sleep group and Brain region was significant [*F* (1, 117) = 12.19, *p* = 0.001, *η*_*P*_^2^ = 0.07]; the post hoc analyses indicated that the participants in insomnia group showed larger alpha power in the posterior regions than participants in the control group (*EMM*_insomnia_ =  − 1.24, *SE* = 0.42; *EMM*_control_ =  − 2.44, *SE* = 0.41, *p* = 0.047), whereas no such difference was revealed in the anterior regions (*p* = 0.59). No other two-way interaction nor three-way interaction effects were revealed (*ps* > 0.05).

The relative power of the theta1 band revealed a significant main effect of Brain region [*F* (1, 117) = 11.78, *p* = 0.001, *η*_*P*_^2^ = 0.09], with the larger theta1 power in the posterior regions than the anterior regions (*EMM*_anterior_ = 1.51, *SE* = 0.16; *EMM*_posterior_ = 1.89, *SE* = 0.16, *p* < 0.001). In addition, no other main nor interaction effects reached significance (*ps* > 0.05).

The relative power of the theta2 band revealed significant main effects of Painful materials and Brain region [*F* (1, 117) = 7.05, *p* = 0.009, *η*_*P*_^2^ = 0.05;* F* (1, 117) = 4.54, *p* = 0.04, *η*_*P*_^2^ = 0.03]. The non-painful images induced larger theta2 power than the painful images (*EMM*_painful_ = 0.96, *SE* = 0.21; *EMM*_non-painful_ = 1.31, *SE* = 0.21, *p* = 0.001), and the larger theta2 power was revealed in the posterior regions than the anterior regions (*EMM*_anterior_ = 1.00, *SE* = 0.30; *EMM*_posterior_ = 1.28, *SE* = 0.30, *p* = 0.04). The interaction effect between Sleep group and Brain region was significant [*F* (1, 117) = 5.68, *p* = 0.02, *η*_*P*_^2^ = 0.04]. The post hoc analyses indicated that participants in the insomnia group showed larger theta2 power in the posterior regions than participants in the control group (*EMM*_insomnia_ = 1.70, *SE* = 0.30; *EMM*_control_ = 0.86, *SE* = 0.29, *p* = 0.049), while the theta2 power did not differ in the anterior regions between two groups (*p* = 0.61). No other two-way interaction nor three-way interaction effects were revealed (*ps* > 0.05).

### Correlation between task performance and time-frequency-domain indicators

**Fig. 7:**
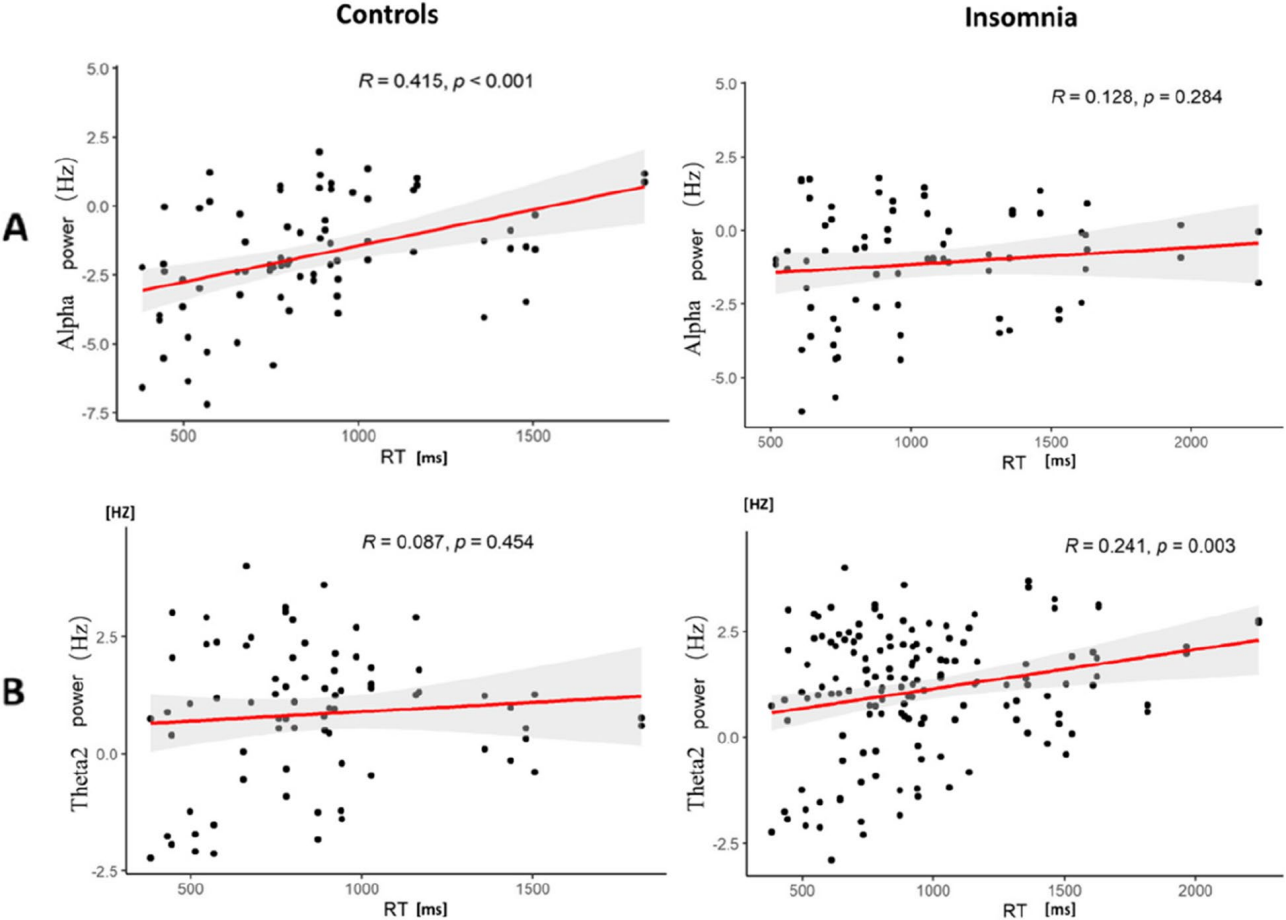
Scatter plot of the correlation coefficient. **A** Scatter plot of alpha frequency band and response time for two groups. **B** Scatter plot of theta2 frequency band and response time for two groups. *Note*: The shaded area indicates the confidence interval estimated from the standard error. The alpha power is the energy intensity after decibel conversion relative to the baseline component and therefore has negative values

As the reported significant Sleep group related interactions for alpha and theta2, thus the correlations between these two bands and RT were calculated. Results showed that the relative power of the alpha band was significantly correlated with RT for painful and non-painful images in the control group (*r* = 0.42, *p* < 0.001) but not in the insomnia group (*r* = 0.13, *p* = 0.29). The relative power of theta2 significantly correlated with RT in the insomnia group (*r* = 0.31, *p* = 0.007) but not in the control group (*r* = 0.09, *p* = 0.45) (see Fig. 7).

## Discussion

In the current study, the behavioral and neurophysiological responses in empathy for pain in individuals with insomnia in non-clinical samples were investigated simultaneously by using the electroencephalogram technique. The findings revealed no statistically significant differences in behavioral performance on empathy for pain task between the insomnia and control groups, whereas individuals with insomnia showed altered time–frequency domain-specific neural activities involved in empathic processing of pain.

The current study employed the empathy for pain task to investigate whether individuals with insomnia showed a difference in perception and emotional response to painful and non-painful materials compared to the healthy. The findings revealed that the subjective rating of painful materials did not significantly differ between the insomnia and control groups. This might be due to the ceiling effect that such easier-to-process painful materials were employed in the current study, and thus, both groups were able to accurately and quickly detect whether the person in images was painful or not. With the painful images being perceived as more painful than the non-painful images in both insomnia and control groups, it could be speculated that the painful images could be easily distinguished from non-painful images and that the current manipulations of the images were valid. One other potential reason for null effects might be due to the relatively young age of the participants [16], and thus, future explorations could illuminate whether age played a critical role in these findings.

By recording the stimulus-locked ERPs, the current findings revealed insomnia-specific changes in early bottom-up attention to painful images, with smaller P2 amplitude to painful images than to non-painful images being showed for the insomnia group but not for the control group. The early components (i.e., P2) were important neural indicators of empathic reactions to other people’s pain and indicated stimulus-driven bottom-up attentional processing [9]. The P2 amplitude represents enhanced attention [42]. Research on empathy for pain in the healthy consistently revealed that P2 is more sensitive to pain stimuli [20, 32]. The reported smaller P2 to painful images versus non-painful images in the insomnia group—however—is consistent with a previous study reporting that non-painful images elicited larger P2 amplitudes than painful images did in sleep-deprived subjects [17]. This could be explained by the fact that painful stimuli which were inherently potentially threatening elicited stronger avoidance motivation and thus shifted more attention resources to non-painful stimuli [43, 44].

Meanwhile, the current findings revealed that both the mean amplitude of N2 and peak amplitude of P2 were positively correlated with the behavioral response time in the control group but not in the insomnia group, suggesting that the increased attention focusing on painful or non-painful stimulus in the early processing stage would delay response time, but this was not the case for individuals with insomnia. This result would also suggest that attentional cues may exert differential modulatory effects on empathy-related processing of pain in individuals with insomnia and healthy individuals.

Besides ERP analyses, the TF analyses were used to explore whether the empathy for pain was differently characterized by frequency domain-specific neural activities between the insomnia group and the control group. The results showed that relatively larger alpha power in posterior brain regions was elicited in the insomnia vs. control group. Alpha power reflects cognitive inhibition and has been considered a reverse activation measure [45]. These findings suggested that participants in the insomnia group showed less cognitive inhibition to painful stimuli compared to those in the control group, and it may reflect a deficiency in the empathic process in individuals with insomnia.

Furthermore, the relatively larger power density of the theta2 band in the posterior brain regions was elicited in participants in the insomnia group than those in the control group. This is consistent with previous findings that an increase in theta power was revealed in adult and adolescent hyperactivity disorder (ADHD) patients when compared to normal controls [46, 47]. The theta oscillation is an indicator of selective voluntary attention [48]. Thus, increased theta2 oscillations may indicate that individuals with insomnia require more attentional resources to process the sharing of emotions in response to others’ pain. Meanwhile, the painful images induced smaller theta2 power than the non-painful images, while no significant difference was revealed on theta1 in the current study, which contrasts with the previous findings reporting stronger theta1 power was elicited by pain images than non-pain images [17]. This discrepancy might be probably due to the differences in sample characteristics and experimental manipulation. More specifically, Duan et al. [17] recruited individuals with normal sleep and manipulated the experimental condition by acute sleep deprivation, while we recruited both individuals with chronic insomnia and healthy controls and compared the characteristics of empathy for pain of both groups. The correlational analyses between the response time of subjective ratings and neural oscillations revealed that the relative power of the alpha band positively correlated with response times in the control group, but the delta2 positively correlated with response time in the insomnia group, suggesting that the alpha band and the theta band may be the possible neural inhibition mechanisms behind the influence of insomnia on empathy for pain to some extent. These findings suggest that the neural manifestation of empathy for pain might change and thus result in behavioral bias for those with chronic insomnia. However, this possibility is tentative and needs to be tested more systematically in the future.

Some limitations need to the mentioned in the current study. Firstly, objective sleep parameters (i.e., sleep architecture, sleep EEG) were not collected in the current study. Thus, the relationship between objective sleep outcomes and the behavioral and neural response to empathy for pain cannot be calculated. Secondly, personal traits such as empathy trait were not assessed in the current study, and whether it would modulate neurobehavioral response to empathy for pain remains unknown. In addition, it should be noted that the interaction on the P2 component was significant only at the peak level, which might be particularly susceptible to noise. Furthermore, the recruited participants in the insomnia group were non-clinical young people, which limits the generalization of the current findings to other populations. One potential reason for the null results found in the current study might be due to the fact that the non-clinical insomnia disorder might not be as severe as clinical insomnia disorder, which might obscure the actual effects of insomnia on empathy for pain. Hence, a re-examination of the current findings in the clinical insomnia disorder group is recommended.

## Conclusion

The current study evidenced that individuals with insomnia do not have a behavioral bias in the subjective rating of pain for painful and non-painful images, whereas the insomnia-specific differences in time-domain and frequency-domain neurophysiological activities are revealed. Insomnia individuals but not the healthy controls showed smaller P2 amplitude to painful versus non-painful images. Additionally, the power in the alpha band and theta2 band in the posterior brain regions was greater for individuals with insomnia. These findings suggested that individuals with insomnia exhibit altered neurophysiological responses to pain stimuli and a diminished ability to empathize for pain, potentially involving changes in attentional mechanisms.

### Supplementary Information

**Additional file 1:** **STable1.** LMM analysis results of peak amplitude of N2 components. **STable2.** LMM analysis results of mean amplitude of N2 components. **STable3.** LMM analysis results of peak amplitude of P2 components. **STable4.** LMM analysis results of mean amplitude of P2 components. **STable5.** LMM analysis results of mean amplitude of LPC components. **STable6.** Descriptive statistical results of ERP components. **STable7.** Descriptive statistical results of Time-frequency-domain indicators.

## Data Availability

The datasets used and/or analyzed during the current study are available from the corresponding authors upon reasonable request.

## Abbreviations

ERP: Event-related potential
LPC: Late positive component
PSQI: Pittsburgh Sleep Quality Index
ISI: Insomnia Severity Index
SAS: Self-rating Anxiety Scale
PANAS: Positive affective and negative affective schedule
M: Mean
SD: Standard deviation
EEG: Electroencephalogram
EOG: Electrooculogram
ICA: Independent component analysis
ICSD: International Classification of Sleep Disorders
MCWT: Morlet continuous wavelet transform
TFRs: Time-frequency representations
TF-ROIs: Time-frequency regions of interest
S-ROIs: Spatial regions of interest
LMM: Linear mixed model
ACC: Accuracy rate
RT: Response time
EMM: Estimated marginal mean
ERD: Event-related desynchronization
ERS: Event-related synchronization
SE: Standard error
