# Supplementary material for: Altered neurophysiological responses during empathy for pain in insomnia: evidence from an EEG study in non-clinical samples
Source: J Physiol Anthropol. 2024 Jan 3;43:4. doi: 10.1186/s40101-023-00351-2 (PMC10765821; doi:10.1186/s40101-023-00351-2)
Supplement: Supplementary file 1 — Additional file 1: STable1. LMM analysis results of peak amplitude of N2 components. STable2. LMM analysis results of mean amplitude of N2 components. STable3. LMM analysis results of peak amplitude of P2 components. STable4. LMM analysis results of mean amplitude of P2 components. STable5. LMM analysis results of mean amplitude of LPC components. STable6. Descriptive statistical results of ERP components. STable7. Descriptive statistical results of Time-frequency-domain indicators. [file 40101_2023_351_MOESM1_ESM.docx]

| STable1. LMM analysis results of peak amplitude of N2 components | | | | |
| --- | --- | --- | --- | --- |
|  | *df* | *F* | *p* | *ηp2* |
| Group | (1,43) | 2.13 | 0.15 | 0.044 |
| Valence | (1,39) | 0.08 | 0.78 | 0.008 |
| Group*Valence | (1,39) | 0.3 | 0.59 | 0.006 |

| STable2. LMM analysis results of mean amplitude of N2 components | | | | |
| --- | --- | --- | --- | --- |
|  | *df* | *F* | *p* | *ηp2* |
| Group | (1,43) | 2.26 | 0.14 | 0.048 |
| Valence | (1,39) | 0.03 | 0.86 | 0.001 |
| Group*Valence | (1,39) | 0.69 | 0.41 | 0.015 |

| STable3. LMM analysis results of peak amplitude of P2 components | | | | |
| --- | --- | --- | --- | --- |
|  | *df* | *F* | *p* | *ηp2* |
| Group | (1,43) | 0.38 | 0.54 | 0.007 |
| Valence | (1,39) | 2.4 | 0.13 | 0.048 |
| Group*Valence | (1,39) | 4.57 | 0.04 | 0.091 |

| STable4. LMM analysis results of mean amplitude of P2 components | | | | |
| --- | --- | --- | --- | --- |
|  | *df* | *F* | *p* | *ηp2* |
| Group | (1,43) | 0.37 | 0.55 | 0.008 |
| Valence | (1,39) | 0.94 | 0.34 | 0.021 |
| Group*Valence | (1,39) | 2.06 | 0.16 | 0.046 |

| STable5. LMM analysis results of mean amplitude of LPC components | | | | |
| --- | --- | --- | --- | --- |
|  | *df* | *F* | *p* | *ηp2* |
| Group | (1,43) | 0.2 | 0.66 | 0.003 |
| Valence | (1,39) | 15.53 | <0.001 | 0.25 |
| Group*Valence | (1,39) | 3.43 | 0.07 | 0.06 |

| STable6. Descriptive statistical results of ERP components | | | | |
| --- | --- | --- | --- | --- |
|  |  | |  |  |
|  | Controls | | Insomnia | |
|  | Non-painful | Painful | Non-painful | Painful |
|  | M±SE | M±SE | M±SE | M±SE |
| N2_mean amplitude | -4.57±0.823 | -4.74±0.823 | -2.83±0.846 | -3.43±0.846 |
| N2_peak amplitude | -4.65±0.72 | -5.10±0.72 | -3.15±0.74 | -3.85±0.74 |
| N2_peak latency | 248±4.83 | 239±4.83 | 252±4.96 | 250±4.96 |
| P2_mean amplitude | -0.994±0.627 | -0.937±0.627 | -0.596±0.644 | -1.020±0.644 |
| P2_peak amplitude | 0.93±0.89 | 1.14±0.89 | 1.71±0.92 | 0.39±0.92 |
| P2_peak latency | 169±3.33 | 169±3.42 | 178±3.33 | 171±3.42 |
| LPC_mean amplitude | 1.79±1.02 | 4.04±1.02 | 1.14±1.05 | 2.43±1.05 |

| STable7. Descriptive statistical results of Time-frequency-domain indicators | | | | |
| --- | --- | --- | --- | --- |
|  |  |  | Controls | Insomnia |
|  |  |  | M±SE | M±SE |
|  | Anterior | Non-painful | -1.11±0.43 | -0.82±0.43 |
| Alpha |  | Painful | -1.17±0.43 | -0.83±0.44 |
|  | Posterior | Non-painful | -2.53±0.43 | -1.22±0.43 |
|  |  | Painful | -2.36±0.44 | -1.27±0.44 |
|  | Anterior | Non-painful | 1.63±0.22 | 1.57±0.22 |
| theta1 |  | Painful | 1.43±0.22 | 1.43±0.22 |
|  | Posterior | Non-painful | 1.23±0.22 | 1.73±0.22 |
|  |  | Painful | 1.13±0.22 | 1.36±0.22 |
|  | Anterior | Non-painful | 1.01±0.32 | 1.39±0.33 |
| theta2 |  | Painful | 0.76±0.32 | 1.82±0.33 |
|  | Posterior | Non-painful | 0.92±0.32 | 1.91±0.33 |
|  |  | Painful | 0.80±0.32 | 1.49±0.33 |
